# Frequency of fatigue and its changes in the first 6 months after traumatic brain injury: results from the CENTER-TBI study

**DOI:** 10.1007/s00415-020-10022-2

**Published:** 2020-07-16

**Authors:** Nada Andelic, Cecilie Røe, Cathrine Brunborg, Marina Zeldovich, Marianne Løvstad, Daniel Løke, Ida M. Borgen, Daphne C. Voormolen, Emilie I. Howe, Marit V. Forslund, Hilde M. Dahl, Nicole von Steinbuechel, CENTER-TBI participants investigators, Cecilia Åkerlund, Krisztina Amrein, Nada Andelic, Lasse Andreassen, Audny Anke, Anna Antoni, Gérard Audibert, Philippe Azouvi, Maria Luisa Azzolini, Ronald Bartels, Pál Barzó, Romuald Beauvais, Ronny Beer, Bo-Michael Bellander, Antonio Belli, Habib Benali, Maurizio Berardino, Luigi Beretta, Morten Blaabjerg, Peter Bragge, Alexandra Brazinova, Vibeke Brinck, Joanne Brooker, Camilla Brorsson, Andras Buki, Monika Bullinger, Manuel Cabeleira, Alessio Caccioppola, Emiliana Calappi, Maria Rosa Calvi, Peter Cameron, Guillermo Carbayo Lozano, Marco Carbonara, Simona Cavallo, Giorgio Chevallard, Arturo Chieregato, Giuseppe Citerio, Iris Ceyisakar, Hans Clusmann, Mark Coburn, Jonathan Coles, Jamie D Cooper, Marta Correia, Amra Čović, Nicola Curry, Endre Czeiter, Marek Czosnyka, Claire Dahyot-Fizelier, Paul Dark, Helen Dawes, Véronique Keyser, Vincent Degos, Francesco Della Corte, Hugo Boogert, Bart Depreitere, Đula Đilvesi, Abhishek Dixit, Emma Donoghue, Jens Dreier, Guy -Loup Dulière, Ari Ercole, Patrick Esser, Erzsébet Ezer, Martin Fabricius, Valery L Feigin, Kelly Foks, Shirin Frisvold, Alex Furmanov, Pablo Gagliardo, Damien Galanaud, Dashiell Gantner, Guoyi Gao, Pradeep George, Alexandre Ghuysen, Lelde Giga, Ben Glocker, Jagoš Golubovic, Pedro A Gomez, Johannes Gratz, Benjamin Gravesteijn, Francesca Grossi, Russell L Gruen, Deepak Gupta, Juanita A Haagsma, Iain Haitsma, Raimund Helbok, Eirik Helseth, Lindsay Horton, Jilske Huijben, Peter J Hutchinson, Bram Jacobs, Stefan Jankowski, Mike Jarrett, Ji -yao Jiang, Faye Johnson, Kelly Jones, Mladen Karan, Angelos G Kolias, Erwin Kompanje, Daniel Kondziella, Evgenios Koraropoulos, Lars -Owe Koskinen, Noémi Kovács, Ana Kowark, Alfonso Lagares, Linda Lanyon, Steven Laureys, Fiona Lecky, Didier Ledoux, Rolf Lefering, Valerie Legrand, Aurelie Lejeune, Leon Levi, Roger Lightfoot, Hester Lingsma, Andrew I.R. Maas, Ana M Castaño-León, Marc Maegele, Marek Majdan, Alex Manara, Geoffrey Manley, Costanza Martino, Hugues Maréchal, Julia Mattern, Catherine McMahon, Béla Melegh, David Menon, Tomas Menovsky, Ana Mikolic, Benoit Misset, Visakh Muraleedharan, Lynnette Murray, Ancuta Negru, David Nelson, Virginia Newcombe, Daan Nieboer, József Nyirádi, Otesile Olubukola, Matej Oresic, Fabrizio Ortolano, Aarno Palotie, Paul M Parizel, Jean -François Payen, Natascha Perera, Vincent Perlbarg, Paolo Persona, Wilco Peul, Anna Piippo-Karjalainen, Matti Pirinen, Horia Ples, Suzanne Polinder, Inigo Pomposo, Jussi P Posti, Louis Puybasset, Andreea Radoi, Arminas Ragauskas, Rahul Raj, Malinka Rambadagalla, Jonathan Rhodes, Sylvia Richardson, Sophie Richter, Samuli Ripatti, Saulius Rocka, Cecilie Roe, Olav Roise, Jonathan Rosand, Jeffrey V Rosenfeld, Christina Rosenlund, Guy Rosenthal, Rolf Rossaint, Sandra Rossi, Daniel Rueckert, Martin Rusnák, Juan Sahuquillo, Oliver Sakowitz, Renan Sanchez-Porras, Janos Sandor, Nadine Schäfer, Silke Schmidt, Herbert Schoechl, Guus Schoonman, Rico Frederik Schou, Elisabeth Schwendenwein, Charlie Sewalt, Toril Skandsen, Peter Smielewski, Abayomi Sorinola, Emmanuel Stamatakis, Simon Stanworth, Robert Stevens, William Stewart, Ewout W. Steyerberg, Nino Stocchetti, Nina Sundström, Anneliese Synnot, Riikka Takala, Viktória Tamás, Tomas Tamosuitis, Mark Steven Taylor, Braden Te Ao, Olli Tenovuo, Alice Theadom, Matt Thomas, Dick Tibboel, Marjolein Timmers, Christos Tolias, Tony Trapani, Cristina Maria Tudora, Peter Vajkoczy, Shirley Vallance, Egils Valeinis, Zoltán Vámos, Mathieu Jagt, Gregory Steen, Joukje Naalt, T.J.M. van Dijck Jeroen, Thomas A. Essen, Wim Hecke, Caroline Heugten, Dominique Praag, Thijs Vyvere, P. J. RoelWijk, Alessia Vargiolu, Emmanuel Vega, Kimberley Velt, Jan Verheyden, Paul M Vespa, Anne Vik, Rimantas Vilcinis, Victor Volovici, Nicole Steinbüchel, Daphne Voormolen, Petar Vulekovic, Kevin K.W. Wang, Eveline Wiegers, Guy Williams, Lindsay Wilson, Stefan Winzeck, Stefan Wolf, Zhihui Yang, Peter Ylén, Alexander Younsi, Frederick A. Zeiler, Veronika Zelinkova, Agate Ziverte, Tommaso Zoerle

**Affiliations:** 1 Department of Physical Medicine and Rehabilitation, Oslo University Hospital, Oslo, Norway; 2 Faculty of Medicine, Institute of Health and Society, Research Centre for Habilitation and Rehabilitation Models and Services (CHARM), University of Oslo, Oslo, Norway; 3 Faculty of Medicine, Institute of Clinical Medicine, University of Oslo, Oslo, Norway; 4 Oslo Centre for Biostatistics and Epidemiology, Oslo University Hospital, Oslo, Norway; 5 Institute of Medical Psychology and Medical Sociology, University Medical Center, Göttingen, Germany; 6 Research Department, Sunnaas Rehabilitation Hospital, Bjørnemyr, Norway; 7 Department of Psychology, Faculty of Social Sciences, University of Oslo, Oslo, Norway; 8 Department of Public Health, Erasmus MC, University Medical Center, Rotterdam, The Netherlands; 9 Department of Child Neurology, Oslo University Hospital, Oslo, Norway

**Keywords:** Head injury, Post-traumatic fatigue, Longitudinal studies, Neurological disorders

## Abstract

**Background:**

Fatigue is one of the most commonly reported subjective symptoms following traumatic brain injury (TBI). The aims were to assess frequency of fatigue over the first 6 months after TBI, and examine whether fatigue changes could be predicted by demographic characteristics, injury severity and comorbidities.

**Methods:**

Patients with acute TBI admitted to 65 trauma centers were enrolled in the study Collaborative European NeuroTrauma Effectiveness Research in TBI (CENTER-TBI). Subjective fatigue was measured by single item on the Rivermead Post-Concussion Symptoms Questionnaire (RPQ), administered at baseline, three and 6 months postinjury. Patients were categorized by clinical care pathway: admitted to an emergency room (ER), a ward (ADM) or an intensive care unit (ICU). Injury severity, preinjury somatic- and psychiatric conditions, depressive and sleep problems were registered at baseline. For prediction of fatigue changes, descriptive statistics and mixed effect logistic regression analysis are reported.

**Results:**

Fatigue was experienced by 47% of patients at baseline, 48% at 3 months and 46% at 6 months. Patients admitted to ICU had a higher probability of experiencing fatigue than those in ER and ADM strata. Females and individuals with lower age, higher education, more severe intracranial injury, preinjury somatic and psychiatric conditions, sleep disturbance and feeling depressed postinjury had a higher probability of fatigue.

**Conclusion:**

A high and stable frequency of fatigue was found during the first 6 months after TBI. Specific socio-demographic factors, comorbidities and injury severity characteristics were predictors of fatigue in this study.

**Electronic supplementary material:**

The online version of this article (10.1007/s00415-020-10022-2) contains supplementary material, which is available to authorized users.

## Introduction

Fatigue is defined as "the awareness of a decreased capacity for mental and/or physical activity, because of an imbalance in the availability, utilization or restoration of resources needed to perform activities" [1]. It is one of the most commonly reported subjective symptoms following traumatic brain injury (TBI). Precise estimates of post-TBI fatigue vary greatly (21–73%) [24], but it consistently exceeds the prevalence of fatigue in the general population (10–20%) [21]. The existing evidence shows that self-reported fatigue decreases over time after TBI, but some patients continue to report persisting fatigue or may even report an increase in fatigue over time [27]. A previous study assessing fatigue pathways over the first year after TBI showed an increase of fatigue after severe TBI (sTBI), stable fatigue after moderate TBI and a reduction of fatigue levels over time after mild TBI (mTBI) [4]. Other studies have suggested that longstanding fatigue is not limited to patients with sTBI, and may be exacerbated or caused by emotional and cognitive symptoms, sleep disturbances, and pain across all injury severities [29, 30].

Premorbid variables such as emotional/mental health problems, personality traits, pre-existing fatigue, and other medical comorbidities may contribute additionally to vulnerability for the development of fatigue following TBI [6, 12]. The association between fatigue and personal factors such as age, gender, and education have been assessed to a lesser extent [6, 16, 27]. Gender differences in prevalence and severity of fatigue have been reported after stroke [20]. However, studies after TBI found inconsistent effects of age and gender [7, 12, 16, 27], whereas higher education was associated with higher levels of fatigue [41].

The majority of previous studies have been conducted with patients after mTBI, and at greatly varying time-points postinjury [24]. Despite a growing body of literature on fatigue after TBI, there is a lack of large-scale studies on longitudinal fatigue changes across both acute clinical care pathways, and injury severity. Such studies are important to increase the knowledge concerning which factors contribute the most to the occurrence and persistence of fatigue, as well as aid the development of preventive efforts and targeted fatigue interventions.

Several scales have been developed for the assessment of different aspects of fatigue for different purposes [5, 24, 40]. These scales often contain numerous questions [18], which may present a burden to the patients when other symptoms and aspects after TBI also need to be assessed. The Rivermead Post-Concussion Symptoms Questionnaire (RPQ) is a self-rated questionnaire assessing the presence and severity of common post-concussion symptoms after TBI [17, 39]. Fatigue is the most frequently affirmed symptom reported in the questionnaire, which renders this item useful to evaluate progress or regression of symptom severity [39]. In factor analysis of the RPQ, fatigue loads either on somatic/physiological symptoms [31] or on emotional/somatic or cognitive symptoms [3], and is strongly associated with limitations in daily functioning [35]. Taken together, the single fatigue item in the RPQ seems to provide a good estimate of the subjective experience of general fatigue after TBI. Therefore, we used it in a large sample of patients from the Collaborative European NeuroTrauma Effectiveness Research in Traumatic Brain Injury (CENTER-TBI) observational study [22].

The aims of this study are:

1. To assess frequency and severity of fatigue at baseline (i.e., at time of study inclusion), 3 and 6 months post-TBI across age, gender, patients’ clinical pathways in the acute phase and severity of injury.
2. To investigate whether socio-demographic factors, injury severity characteristics, and pre- and postinjury comorbidities predict fatigue changes across the first 6 months following TBI.

We hypothesize that fatigue presents a significant burden for the majority of patients after TBI regardless of injury severity and time since injury.

## Methods

### Study design

Patients were selected from the core study of the CENTER-TBI project; a multicenter, prospective observational longitudinal cohort study, conducted in Europe and Israel [22], which enrolled patients with all severities of TBI who presented to 65 participating centers between December 19, 2014 and December 17, 2017. Inclusion criteria were a clinical diagnosis of TBI, an indication for CT scanning, presenting to a medical center within 24 h of injury, and obtained informed consent adhering to local and national ethical and legal requirements. Patients were excluded if there was a severe pre-existing neurological disorder that could potentially bias outcome assessments (in this study self-reported fatigue). Three strata were used to prospectively differentiate patients by clinical care pathway: emergency room (ER; patients evaluated in the ER and discharged afterwards), admission (ADM; patients admitted to a hospital ward) and intensive care unit (ICU; patients who were primarily admitted to the ICU). The main descriptive findings of CENTER-TBI have been published elsewhere [34].

### Study participants

In total, 4509 participants were enrolled in the CENTER-TBI core study. In the current study, all patients from the ER, ADM and ICU strata who answered the RPQ-fatigue question at least once at either baseline (mean 2.5 days following admission to CENTER-TBI), 3 or 6 months after injury were selected. Thus, 3354 patients (78% of all included in the core study) were included in this study and their baseline characteristics are described in Table 1. Among these, 2286 had answered the RPQ-fatigue question at baseline, 2164 at 3 months after injury, and 2253 at 6 months after injury and were thus further analyzed in this study.

**Table 1:** Characteristics of the study population

Characteristics	Total (N = 3354)	ER (*n* = 808)	ADM (*n* = 1351)	ICU (*n* = 1195)	*p* value
Gender, male %	2189 (65.3%)	449 (55.6%)	877 (64.9%)	863 (72.2%)	< 0.001
Age, years					< 0.001
Mean (SD)	47.8 (21.0)	47.9 (20.7)	50.6 (21.6)	44.6 (20.0)	
Median (IQR)	49 (29, 65)	48 (29, 64)	53 (32, 67)	45 (27, 60)	
Age categories, %					< 0.001
0–18 years	259 (7.7%)	42 (5.2%)	102 (7.5%)	115 (9.6%)	
19–40 years	1040 (31.0%)	280 (34.7%)	357 (26.4%)	403 (33.7%)	
41–65 years	1258 (37.5%)	295 (36.5%)	498 (36.9%)	465 (38.9%)	
> 65 years	797 (23.8%)	191 (23.6%)	394 (29.2%)	212 (17.7%)	
Education, years					0.041
Mean (SD)	13.2 (4.2)	13.1 (4.1)	13.4 (4.3)	13.0 (4.2)	
Median (IQR)	13 (11, 16)	13 (11, 16)	13 (11, 16)	13 (11, 16)	
Employment, %					< 0.001
Working ≥ 35 h/week	1319 (39.3%)	329 (40.7%)	467 (34.6%)	523 (43.8%)	
Working < 35 h/week	310 (9.2%)	89 (11.0%)	127 (9.4%)	94 (7.9%)	
Student	408 (12.2%)	86 (10.6%)	161 (11.9%)	161 (13.5%)	
Retired	793 (23.6%)	199 (24.6%)	375 (27.8%)	219 (18.3%)	
Not working	524 (15.6%)	105 (13.0%)	221 (16.4%)	198 (16.6%)	
Preinjury ASA-PS					< 0.001
Healthy	1991 (59.9%)	462 (57.4%)	758 (56.6%)	771 (65.4%)	
Mild disease	1038 (31.2%)	258 (32.0%)	457 (34.1%)	323 (27.4%)	
Severe disease	293 (8.8%)	85 (10.6%)	124 (9.3%)	84 (7.1%)	
Preinjury Psychiatry	415 (12.9%)	116 (15.1%)	154 (11.8%)	145 (12.5%)	0.088
Previous TBI	(*n* = 3206) 329 (10.3%)	113 (14.5%)	135 (10.3%)	81 (7.2%)	< 0.001
Cause of injury					< 0.001
Traffic accident	1247 (39.1%)	257 (32.9%)	446 (34.6%)	544 (48.6%)	
Incidental fall	1531 (48.0%)	400 (51.3%)	664 (51.6%)	467 (41.7%)	
Others	410 (12.9%)	123 (15.8%)	178 (13.8%)	109 (9.7%)	
GCS categories, %					< 0.001
GCS 13–15	2616 (80.2%)	794 (99.6%)	1285 (97.1%)	537 (47.1%)	
GCS 9–12	221 (6.8%)	2 (0.3%)	32 (2.4%)	187 (16.4%)	
GCS 3–8	424 (13.0%)	1 (0.1%)	6 (0.5%)	417 (36.5%)	
AIS head (≥ 3), %	2094 (63.0%)	64 (7.9%)	946 (70.5%)	1084 (92.2%)	< 0.001
ISS, median (IQR)	13 (8, 25)	4 (2, 8)	10 (9, 17)	26 (18, 41)	< 0.001
CT head—presence of intracranial injury	1359 (42.2%)	71 (9.3%)	469 (36.6%)	819 (70.8%)	< 0.001

*SD* standard deviation; *IQR* interquartile range;* ASA-PS* American Society of Anesthesiologists Physical Status Classification System score; *GCS* Glasgow Coma Scale; *AIS* abbreviated injury severity score; *ISS* injury severity score

### Measurements

Both adults (age group ≥ 16 years) and children and/or their parents (age group < 16 years) were asked to rate the severity of fatigue compared to their preinjury status during the last 24 h. Rating on a 5-point Likert scale was used, from 0 = “not a problem” to 4 = “severe problem”. A study assessing validity showed that RPQ was unbiased for an age range of 6–96 years [19], and parents ratings of fatigue in children with TBI have been applied in research previously [10].

The data were either collected in face-to-face interviews, or per postal or electronic questionnaires at baseline, (mean 2.5 days following study admission, SD ± 12.0), at 3 and at 6 months follow-ups. The cut-off value ≥ 2, corresponding to symptoms rated as mild, moderate and severe, was used as one of the options of evaluation of symptom severity [38]. However, in clinical practice, a sub-group of patients with moderate and/or severe fatigue symptoms may be challenging to treat because of its impact on general functioning and daily activities; thus, a cut-off value ≥ 3, corresponding to symptoms rated as moderate and severe was also applied.

Socio-demographic and injury-related characteristics that were collected at the time of study admission and used as independent variables included gender (female/male), age (continuous, and categorical: 0–18, 19–40, 41–64, > 65 years, and dichotomized at median value) and education (continuous, i.e. in years, and dichotomized at median value).

Preinjury somatic comorbidities were measured by the pre-injury American Society of Anesthesiologists Physical Status Classification System score (ASA-PS) [23].

Preinjury psychiatric conditions comprised anxiety, depression, sleep disorders, schizophrenia, drug abuse or other psychiatric problems as reported by patients retrospectively at follow-up.

Injury-related variables were: injury mechanism (road traffic accident, falls, others); injury severity measured by patient strata, Glasgow Coma Scale (GCS) score/category within the first 24 h after injury [36], presence of intracranial injuries on first CT head, Abbreviated Injury Scale head (AIS head, score ≥ 3 considered as severe intracranial injury) [15], and Injury Severity Score (ISS), where a score > 15 was considered as major overall trauma [2].

Two additional items from RPQ were used to assess sleep disturbances and feeling depressed at baseline, and were applied as determinants of postinjury comorbidities of potential relevance for feeling fatigued. A cut-off score of ≥ 2 (mild, moderate and severe problems) was used.

### Statistical analysis

The CENTER-TBI dataset version 2.0 (dataset from May 2019) was analyzed in this manuscript. The frequency of patients experiencing fatigue was assessed per patient strata, age group, gender and GCS severity level.

For descriptive statistics means with standard deviations (SD), medians with interquartile range (IQR), or percentages are presented. Differences in demographic and injury related data between patients’ strata ER, ADM and ICU were tested using a one-way ANOVA or Kruskal–Wallis test for continuous variables. A chi-square test for contingency tables was performed to detect group differences in categorical variables.

To analyze changes in fatigue between the patients’ strata over the entire follow-up period and account for repeated measures by patient, mixed effect logistic regression was performed using fatigue (dichotomized at the value ≥ 2) as the outcome variable. Time and time-by-patient strata interaction were introduced as fixed effects in all models. Based on the mixed effects logistic regression, we estimated risk differences with 95% confidence intervals (CI) from baseline to 6 months using the delta method. For comparison of the effects of different cut-offs, the analysis was replicated using fatigue dichotomized at the value ≥ 3 as the outcome variable.

Further, mixed effect logistic regression analyses were performed to investigate whether changes of fatigue (dichotomized at the value ≥ 2/ ≥ 3) during the follow-up period (baseline, 3, and 6 months) could be predicted by age, gender, patient strata, education, preinjury ASA-PS and psychiatric comorbidities, GCS score, intracranial injury on CT, AIS head, ISS, and RPQ items `feeling depressed`, and `sleep disturbance` (dichotomized at the value of ≥ 2). Time and all predictor variables were treated as fixed effects in the models. Interaction effects between time and fixed factors were verified by introducing product terms. All models included a random intercept. Statistically significant fixed main effects or interaction effects on fatigue ≥ 2 were graphed across each of the three time points. In these figures, if the predictor was continuous a median-split procedure was used to generate separate lines as function of the predictor.

Missing predictor data were handled by multiple imputations with ten imputations applying the Markov Chain Monte Carlo method [32]. Sensitivity analyses were performed to handle missing values in predictor variables. The multiple imputed model was compared with the complete case analyses, and presented in results.

All statistical analyses were performed using IBM SPSS Statistics for Windows version 25 (Armonk, NY: IBM Corp.) and Stata 15 (Stata Corp LLC, College Station, TX).

## Results

Table 1 shows demographic and injury characteristics by patient strata; 808 patients were included in the ER stratum, 1351 in ADM, and 1195 in ICU. Median age of the total sample was 49 (IQR 29, 65) years and 65% of the participants were male. Median years of education was 13 (IQR 11, 16) years. Socio-demographics and injury severity characteristics differed significantly between patient strata **(**Table 1**)**. Severe TBI (GCS 3–8), severe intracranial injury (AIS head ≥ 3) and severe overall trauma (ISS > 15) were observed in 37, 92 and 95% of patients in ICU stratum, respectively.

Furthermore, 2286 patients reported on the fatigue item at baseline and were thus evaluated in this study. Of these, 46.9% reported having fatigue (cut-off score ≥ 2). The frequency was halved when using moderate/severe fatigue cut-off score (≥ 3) (22.8%). The median fatigue score was highest in the patients admitted to ICU (2, IQR 0–3, *p* = 0.001) where 57.6% reported moderate/severe fatigue. In ADM and ER strata, 48.2 and 39.0% participants experienced moderate/severe fatigue, respectively (Table 2)**.**

**Table 2:** Fatigue severity scores at baseline by patient strata

Fatigue scores at baseline	Total (*n* = 2286)	ER (*n* = 745)	ADM (*n* = 1142)	ICU (*n* = 399)	*p* value
Median (IQR)	1 (0, 2)	0 (0, 2)	1 (0, 2)	2 (0, 3)	< 0.001
Severity of fatigue					< 0.001
None (0–1)	1215 (53.1%)	454 (60.9%)	592 (51.8%)	169 (42.4%)	
Mild problem (2)	549 (24.0%)	160 (21.5%)	285 (25.0%)	104 (21.6%)	
Moderate or severe problem (3–4)	522 (22.8%)	131 (17.6%)	265 (23.2%)	126 (31.6%)	
Fatigue scores ≥ 2	1071 (46.9%)	291 (39.1%)	550 (48.2%)	230 (57.6%)	< 0.001

*ER* emergency room; *ADM* admission; *ICU* intensive care unit; *IQR* interquartile range

eTable 1 in the Supplement presents fatigue scores by age groups and patients’ strata. In the ER stratum, the highest prevalence of moderate/severe fatigue was in the age group 19–40 (22.4%); in the ADM stratum in the age group 0–18 (34.9%). The most frequently reported moderate/severe fatigue was in the ICU stratum in age group 0–18 (48.8%), and age groups 19–40 and 41–65 years (32.4 and 31.4%, respectively).

**Fig. 1:**
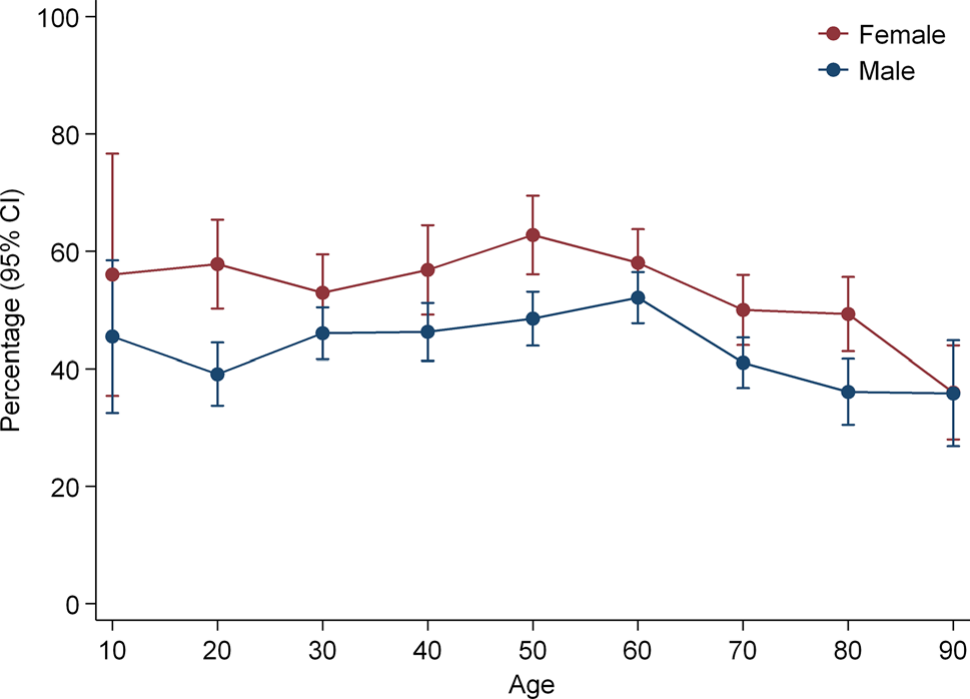
Frequency of patients with Fatigue (≥ 2) by 10-year age groups and gender at study admission

The frequency of fatigue by 10-year age groups and gender is presented in Fig. 1. Overall, 52.5% of females and 43.6% of males reported fatigue; the frequency was highest in females across all age groups. The highest frequency of moderate/severe fatigue (≥ 3) was found for females aged 50–60 years (38.3%) and males aged 0–10 years (46.4%), and the lowest in females aged 60–70 years (20.3%) and males > 70 years (8.5%).

### Changes of fatigue across 6 months follow-up

**Fig. 2:**
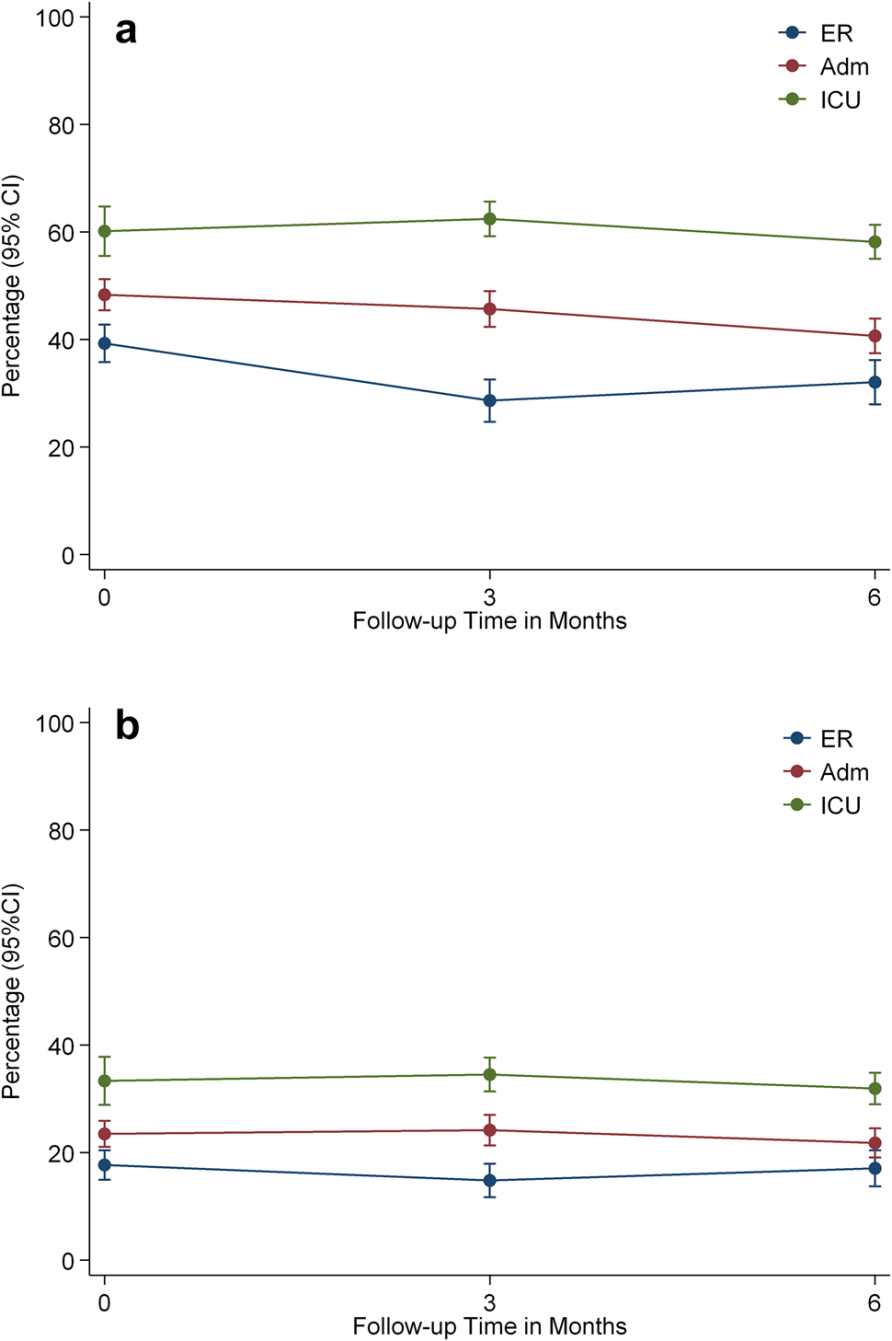
**a** Estimated proportions of patients with Fatigue ≥ 2 by patient strata. **b** Estimated proportions of patients with Fatigue ≥ 3 by patient strata

The estimated proportions of fatigue score ≥ 2 and ≥ 3 by patients strata are reported in Fig. 2a, b.

Overall, there were no statistically significant differences in fatigue proportions between patient strata`s across the first 6 months post injury. However, significant within group differences due to a decrease in fatigue scores ≥ 2 were found in the ER (mean change − 7.2, 95%CI − 12.0 to − 2.4, *p* = 0.003) and ADM (mean change: − 7.7, 95% CI − 11.5 to − 3.8, *p* < 0.001) strata from baseline to 6 months*,* but not for the ICU group (mean change − 2.0, 95%CI − 7.2 to 3.2, *p* = 0.454). When applying cut-off ≥ 3, representing moderate and severe fatigue, no such reduction was observed, indicating more persistence of severe symptoms compared to mild.

Similar results were found in the modeling of changes of fatigue scores ≥ 2 and the score ≥ 3 by GCS severity categories supporting the notion that the clinical pathways in the acute TBI phase are indicators of injury severity **(**eFigures 1 a and 1b and eTable 2 in the Supplement)*.*

### Predictors of fatigue changes

Two models used in the predictive analyses examined whether changes of fatigue scores ≥ 2 (model 1) and ≥ 3 (model 2) over time could be predicted by demographic variables, injury severity indicators and comorbidities. All statistically significant and non-significant fixed effects from the full model and their coefficients, p-values, and 95% confidence intervals are presented in Table 3.

**Table 3:** Predictors of fatigue (imputed predictors)

	Model 1			Model 2		
	Coef	95% CI	*p* value	Coef	95% CI	*p* value
Intercept	− 0.83***	− 1.43 to − 0.22	0.007	− 2.21	− 2.88 to − 1.55	< 0.001
Time	− 0.18	− 0.31 to − 0.04	0.012	− 0.04	− 0.20 to 0.11	0.596
Patient strata						
ER	Ref					
Adm	0.30	− 0.02 to 0.62	0.070	0.16	− 0.23 to 0.54	0.425
ICU	0.61**	0.13 to 1.09	0.013	0.45	− 0.10 to 0.99	0.109
Age, y	− 0.02***	− 0.03 to − 0.02	< 0.001	− 0.02***	− 0.03 to − 0.01	< 0.001
Gender (f = 0, m = 1)	− 0.62***	− 0.86 to − 0.38	< 0.001	− 0.60***	− 0.87 to − 0.33	< 0.001
Education, y	0.05**	0.02 to 0.07	0.001	0.04*	0.01 to 0.07	0.007
Preinjury ASA-PS						
Healthly patients	Ref					
Mild disease	0.28*	0.004 to 0.56	0.047	0.19	− 0.13 to 0.51	0.244
Severe disease	0.47*	0.03 to 0.91	0.034	0.55*	0.06 to 1.04	0.028
Preinjury psychiatry	0.12	− 0.23 to 0.47	0.491	0.20	− 0.19 to 0.58	0.321
GCS (3–15)	0.08	− 0.19 to 0.35	0.565	0.05	− 0.23 to 0.33	0.727
CT head intracranial injury	0.08	− 0.20 to 0.36	0.577	0.01	− 0.30 to 0.32	0.961
AIS head (≥ 3)	0.35*	0.03 to 0.67	0.034	0.54**	0.17 to 0.91	0.004
ISS	0.02*	0.00004 to 0.03	0.049	0.02*	0.00002 to 0.03	0.050
Feeling depressed at baseline	1.26***	0.94 to 1.57	< 0.001	1.55***	1.08 to 2.02	< 0.001
Sleep disturbance at baseline	1.18***	0.91 to 1.45	< 0.001	1.82***	1.47 to 2.18	< 0.001
Time × Significant predictors						
Time × ICU	0.04	− 0.08 to 0.15	0.537	0.04	− 0.09 to 0.17	0.568
Time × Age	0.005***	0.003 to 0.01	< 0.001	0.004***	0.002 to 0.01	< 0.001
Time × Gender	− 0.01	− 0.06 to 0.05	0.811	− 0.01	− 0.07 to 0.05	0.666
Time × Education	− 0.01*	− 0.01 to -0.002	0.014	− 0.01*	− 0.02 to − 0.002	0.009
Time × Preinjury ASA-PS						
Time × Mild disease	− 0.01	− 0.07 to 0.05	0.747	0.01	− 0.06 to 0.08	0.743
Time × Severe disease	0.02	− 0.08 to 0.13	0.654	− 0.004	− 0.11 to 0.11	0.942
Time × Preinjury psychiatry	0.12**	0.04 to 0.20	0.004	0.09*	0.0001 to 0.18	0.050
Time × AIS head	0.01	− 0.07 to 0.09	0.788	− 0.04	− 0.13 to 0.05	0.336
Time × ISS	0.0004	− 0.003 to 0.004	0.821	− 0.001	− 0.004 to 0.003	0.601
Time × Feeling Depressed	− 0.16***	− 0.23 to − 0.09	< 0.001	− 0.26***	− 0.37 to − 0.14	< 0.001
Time × Sleep disturbance	− 0.15***	− 0.21 to − 0.08	< 0.001	− 0.22***	− 0.31 to − 0.14	< 0.001

*ER* emergency room; *ADM* admission; *ICU* intensive care unit; *ASA-PS* American Society of Anesthesiologists Physical Status Classification System score; *GCS* Glasgow Coma Scale; *AIS* abbreviated injury severity score; *ISS* injury severity score.

Model 1: Fatigue cut-off ≥ 2, Model 2: Fatigue cut-off ≥ 3. * = *p* < 0.05; ** = *p* < 0.01; *** = *p* < 0.001

In model 1, the ICU patient stratum, age, gender, education, preinjury ASA-PS, AIS head, ISS, feeling depressed, and sleep disturbance yielded significant effects on fatigue probability changes. Patients admitted to ICU had a higher probability of experienced fatigue than those admitted to ER and ADM strata. In addition, patients with lower age, higher education, more severe injuries as assessed by AIS head and ISS, with pre-injury somatic and psychiatric diseases and postinjury comorbidity (sleep disturbance and feelings of depression) and females had a higher probability of fatigue.

**Fig. 3:**
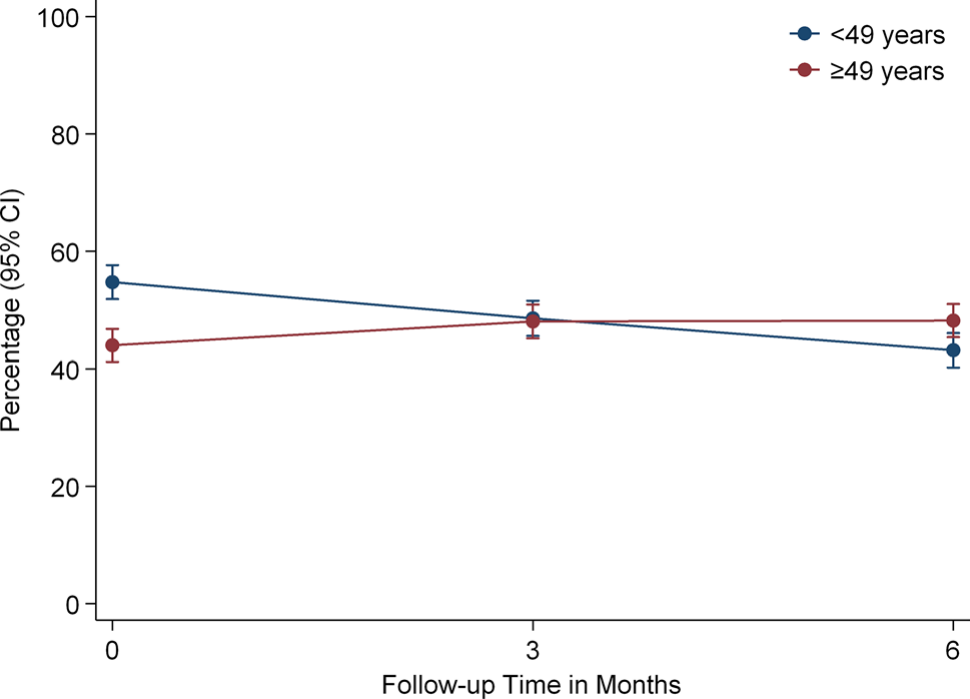
Main effect and time interaction of age on fatigue changes

The significant interaction effect between time and age suggested that the patient group < 49 years tended to report higher fatigue scores initially and then decreased over time, e.g. reported less fatigue, whereas patients ≥ 49 years reported less fatigue symptoms initially and then fatigue slightly increased over time (Fig. 3).

**Fig. 4:**
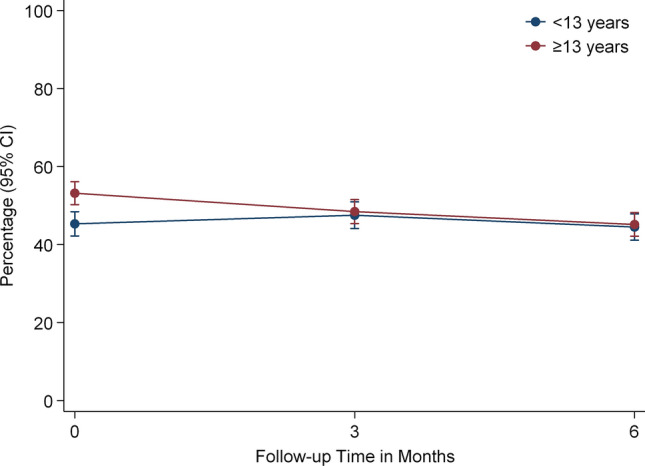
Main effect and time interaction of education on fatigue changes

The significant interaction effect between time and education suggested that patients with higher education (≥ 13 years) tended to report higher fatigue scores initially and then decreased over time, whereas those with lower education reported less fatigue initially, and then slightly higher fatigue scores during the first 3 months (Fig. 4).

**Fig. 5:**
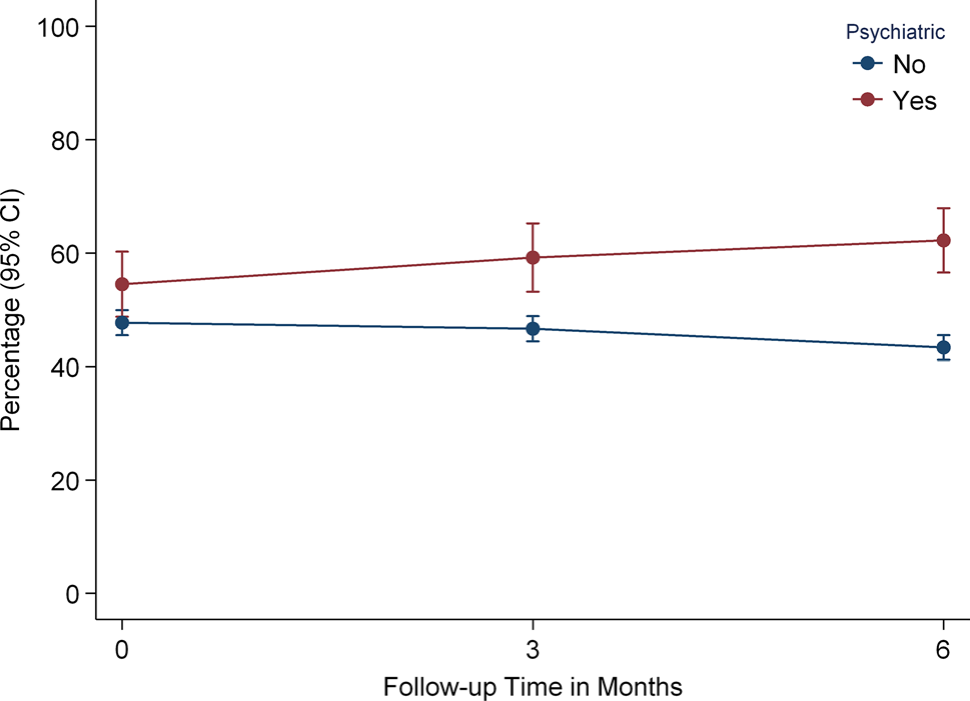
Time interaction of preinjury psychiatric comorbidity on fatigue changes

The significant interaction effect between time and preinjury psychiatric conditions suggested that patients with known psychiatric problems tended to report higher fatigue scores at baseline and then slightly increased scores over time, whereas those without psychiatric conditions reported decreased scores over time (Fig. 5).

The significant interaction effects between time and feeling depressed and sleep disturbance suggested that patients who reported feeling depressed and sleep disturbance (cut-off ≥ 2) tended to report higher fatigue scores initially, then less over the next 3 months and stable levels during the last 3 months. (eFigures 2 and 3 in the Supplement)*.*

In model 2, the same predictors were statistically significant as in model 1 (except the ICU stratum) indicating that the assessed fatigue predictors are of major importance across all fatigue severity levels.

## Discussion

This large-scale, observational longitudinal study assessed the frequency of fatigue following TBI, fatigue changes across clinical care pathways, severity of injury, and predictors of fatigue severity levels.

Fatigue is a widespread symptom in the acute and post-acute TBI phase [39]. As expected, we found a high frequency of fatigue throughout the whole sample included in this study: around 47% of patients reported subjective fatigue of any severity (cut-off ≥ 2) at baseline, 48% at 3 months and 46% at 6 months. These frequencies were halved when cut-off ≥ 3 (moderate and severe fatigue) was used. Females and patients of younger age (≤ 40 years) reported higher frequency of fatigue at baseline. The frequency of fatigue was highest in the patients admitted to the ICU, those with moderate and severe TBI, and more severe intracranial injuries and overall trauma. Our results suggest that more severe TBI may increase the risk of fatigue probably due to the neuro-morphological brain damage as discussed later. However, this is in contrast with previous research that reports no increased risk of fatigue in those with more severe TBI [24].

In line with our expectations, level of fatigue stayed quite stable over the first 6 months post-TBI, particularly, the moderate and severe levels (fatigue cut-off ≥ 3). As fatigue has an unfavorable effect on participation in activities of daily life [4], the results indicate that we should identify those with higher levels of fatigue early after the injury, and provide further assessments, timely advices, and targeted rehabilitation programs.

Demographic factors such as age, gender, and education were associated with fatigue levels in this study. As mentioned previously, findings regarding the association between fatigue following TBI and demographic factors are inconsistent in the literature. For example, Cantor et al. [7] did not find any association between age, gender, education and fatigue. In our study, lower age was associated with higher levels of fatigue, probably reflecting the TBI severity in this population (33% of patients in age group ≤ 40 years had severe TBI, in contrast to 20% of patients in age group > 40 years).

We found that females reported greater levels of fatigue compared to males, in line with previous studies [12]. In studies on self-reported symptoms following TBI, women are more likely to report problems across different symptom domains [14]. Furthermore, post-concussion symptoms and especially fatigue is prevalent in the general population as well [37]. However, previous research has suggested that gender differences in socialization and gender-role expectations may change over time and moderate the relationship between gender and outcome measures after TBI [9, 25].

We also found an association between higher levels of education and greater severity of fatigue, which is in line with study by Ziino & Ponsford [41]. This may relate to a trend in the general population where people with higher education report more symptoms, possibly related to them having a better understanding of health problems and health care services utilization [11]. Another possible explanation may be related to the concept of cognitive reserve, i.e. the fact that education seems to contribute to higher levels of cognitive functioning throughout the life-span, which again may result in individuals with higher education coping better with TBI-related cognitive impairments. However, as people with higher levels of education often work in cognitively demanding professions, the subjective experience of fatigue may hamper the use of cognitive reserves, causing fatigue to feel relatively more detrimental to these persons. Given the mixed results in the current literature regarding the association between education and fatigue levels, future studies on the relationship between education, cognitive reserve and fatigue after TBI are needed.

Furthermore, the present results support a relationship between fatigue and more severe TBI and overall trauma. This was indicated by several significant predictors including the ICU stratum, AIS head ≥ 3 and higher ISS score, all affecting the fatigue levels in this study. Some studies have indicated that post-TBI fatigue was positively associated with greater severity of injury [33] whereas others have failed to demonstrate an association between fatigue and injury severity [24, 28, 41]. Methodological differences between studies may explain these discrepancies. Still, it is worth mentioning that previous studies have suggested that intracranial injuries such as traumatic axonal injury (TAI), global and regional thalamic morphometric changes and functional connectivity in the thalamus and middle frontal cortex may contribute to fatigue following TBI [8, 13, 26]. However, there are only few studies on this topic, and further research on the association between neuro-morphological brain injury and fatigue following TBI is needed.

Presence of preinjury (i.e. somatic disease and psychiatric conditions) and postinjury comorbidities (i.e., feeling depressed and sleep disturbance) also predicted fatigue levels. Participants with preinjury psychiatric conditions, those with depressive feelings and sleep problems were at risk of unfavorable fatigue outcomes in this study. Previous TBI studies with mixed severity samples [6, 12] have demonstrated the association between these comorbidities and fatigue. This is of importance to the field of rehabilitation given the impact these symptoms may have on daily activity levels and health-related quality of life. Treating the symptoms that co-occur with and interact with fatigue such as premorbid psychiatric problems, ongoing depression, sleep problems, and pain and finding a balance between rest and activities (i.e., pacing) is currently the best recommendations for fatigue treatment [30].

Overall, the same factors predicted fatigue regardless of the cut-off (≥ 2 or ≥ 3) applied, indicating the reliability of predictors used in the study. Time since injury interacts with a range of predictors, but does not predict changes on its own, whereas injury severity appears to be a robust predictor. The study findings may help health professionals to plan individualized therapy and rehabilitation programs in the early stages of recovery for patients with specific demographic and injury characteristics and comorbidities.

## Limitations

These findings may not be generalizable to all European individuals who have sustained a TBI since participants were mainly recruited from trauma referral centers. As such, the findings are not necessarily generalizable to individuals sustaining a minimal TBI or a mild TBI without indication for a CT head. One of the major limitations of this study is the use of a single item operationalization of fatigue; nevertheless, it was the only opportunity to measure fatigue and its changes when using the CENTER-TBI data. The wording of the item asks whether fatigue has been a problem for the past 24 h compared to before the injury. The experience of symptoms, however, can vary, and may be related to the level of activity at the time of assessment. This raises the possibility that the reported ratings of fatigue symptoms are not reflective of the overall experience (i.e., both over- and underreporting possible). Using fatigue assessment instruments with established validity in specific patient groups is recommended [40]; yet, such instruments were not available in this study. Further, usage of specific fatigue tools may not be as achievable in a hectic clinical setting as the broad current use of the RPQ, thus our results may be more easily transferrable to common clinical practice.

Fatigue after TBI has increasingly been conceptualized as a complex condition, with a number of factors that may contribute to its development and persistence [30]. Variables included in our predictive models were selected based on clinical importance and previous studies on TBI. Additionally, other variables such as preinjury fatigue symptoms, neurocognitive function, structural brain abnormalities, potential blood biomarkers, and hormonal imbalance not included in this study should be assessed in future studies. Taken together, translational research is needed to advance a clinical decision-making process and targeted medical treatment of fatigue in the future.

## Electronic supplementary material

Below is the link to the electronic supplementary material.

Supplementary file1 (DOCX 67 kb)

## References

[1] Aaronson LS, Teel CS, Cassmeyer V, Neuberger GB, Pallikkathayil L, Pierce J, Press AN, Williams PD, Wingate A (1999). Defining and measuring fatigue. Image J Nurs Sch.

[2] Baker SP, O'Neill B, Haddon W, Long WB (1974). The injury severity score: a method for describing patients with multiple injuries and evaluating emergency care. J Trauma.

[3] Barker-Collo S, Theadom A, Starkey N, Kahan M, Jones K, Feigin V (2018). Factor structure of the Rivermead post-concussion symptoms questionnaire over the first year following mild traumatic brain injury. Brain Inj.

[4] Beaulieu-Bonneau S, Ouellet MC (2017). Fatigue in the first year after traumatic brain injury: course, relationship with injury severity, and correlates. Neuropsychol Rehabil.

[5] Borgaro SR, Baker J, Wethe JV, Prigatano GP, Kwasnica C (2005). Subjective reports of fatigue during early recovery from traumatic brain injury. J Head Trauma Rehabil.

[6] Cantor JB, Ashman T, Gordon W, Ginsberg A, Engmann C, Egan M, Spielman L, Dijkers M, Flanagan S (2008). Fatigue after traumatic brain injury and its impact on participation and quality of life. J Head Trauma Rehabil.

[7] Cantor JB, Bushnik T, Cicerone K, Dijkers MP, Gordon W, Hammond FM, Kolakowsky-Hayner SA, Lequerica A, Nguyen M, Spielman LA (2012). Insomnia, fatigue, and sleepiness in the first 2 years after traumatic brain injury: an NIDRR TBI model system module study. J Head Trauma Rehabil.

[8] Clark AL, Sorg SF, Holiday K, Bigler ED, Bangen KJ, Evangelista ND, Bondi MW, Schiehser DM, Delano-Wood L (2018). Fatigue is associated with global and regional thalamic morphometry in veterans with a history of mild traumatic brain injury. J Head Trauma Rehabil.

[9] Colantonio A, Harris JE, Ratcliff G, Chase S, Ellis K (2010). Gender differences in self reported long term outcomes following moderate to severe traumatic brain injury. BMC Neurol.

[10] Crichton A, Anderson V, Oakley E, Greenham M, Hearps S, Delzoppo C, Beauchamp MH, Hutchison JS, Guerguerian AM, Boutis K, Babl FE (2018). Fatigue following traumatic brain injury in children and adolescents: a longitudinal follow-up 6 to 12 months after injury. J Head Trauma Rehabil.

[11] Cutler DM, Lleras-Muney A (2010). Understanding differences in health behaviors by education. J Health Econ.

[12] Englander J, Bushnik T, Oggins J, Katznelson L (2010). Fatigue after traumatic brain injury: association with neuroendocrine, sleep, depression and other factors. Brain Inj.

[13] Esbjörnsson E, Skoglund T, Sunnerhagen KS (2013) Fatigue, psychosocial adaptation and quality of life one year after traumatic brain injury and suspected traumatic axonal injury; evaluations of patients and relatives. A pilot study. J Rehabil Med in press 10.2340/16501977-1170 24002313

[14] Farace E, Alves WM (2000). Do women fare worse: a metaanalysis of gender differences in traumatic brain injury outcome. J Neurosurg.

[15] Gennarelli TA, Wodzin E (2006). AIS 2005: a contemporary injury scale. Injury.

[16] Juengst SB, Nabasny A, Terhorst L (2019). Neurobehavioral symptoms in community-dwelling adults with and without chronic traumatic brain injury: differences by age, gender, education, and health condition. Front Neurol.

[17] King NS, Crawford S, Wenden FJ, Moss NE, Wade DT (1995). The Rivermead post concussion symptoms questionnaire: a measure of symptoms commonly experienced after head injury and its reliability. J Neurol.

[18] Krupp LB, LaRocca NG, Muir-Nash J, Steinberg AD (1989). The fatigue severity scale. Application to patients with multiple sclerosis and systemic lupus erythematosus. Arch Neurol.

[19] Lannsjo M, Borg J, Bjorklund G, Af Geijerstam JL, Lundgren-Nilsson A (2011). Internal construct validity of the Rivermead post-concussion symptoms questionnaire. J Rehabil Med.

[20] Lerdal A, Bakken LN, Rasmussen EF, Beiermann C, Ryen S, Pynten S, Drefvelin AS, Dahl AM, Rognstad G, Finset A, Lee KA, Kim HS (2011). Physical impairment, depressive symptoms and pre-stroke fatigue are related to fatigue in the acute phase after stroke. Disabil Rehabil.

[21] Lerdal A, Wahl A, Rustoen T, Hanestad BR, Moum T (2005). Fatigue in the general population: a translation and test of the psychometric properties of the Norwegian version of the fatigue severity scale. Scand J Public Health.

[22] Maas AI, Menon DK, Steyerberg EW, Citerio G, Lecky F, Manley GT, Hill S, Legrand V, Sorgner A (2015). Collaborative European NeuroTrauma Effectiveness Research in Traumatic Brain Injury (CENTER-TBI): a prospective longitudinal observational study. Neurosurgery.

[23] Mayhew D, Mendonca V, Murthy BVS (2019). A review of ASA physical status—historical perspectives and modern developments. Anaesthesia.

[24] Mollayeva T, Kendzerska T, Mollayeva S, Shapiro CM, Colantonio A, Cassidy JD (2014). A systematic review of fatigue in patients with traumatic brain injury: the course, predictors and consequences. Neurosci Biobehav Rev.

[25] Niemeier JP, Perrin PB, Holcomb MG, Rolston CD, Artman LK, Lu J, Nersessova KS (2014). Gender differences in awareness and outcomes during acute traumatic brain injury recovery. J Womens Health (Larchmt ).

[26] Nordin LE, Moller MC, Julin P, Bartfai A, Hashim F, Li TQ (2016). Post mTBI fatigue is associated with abnormal brain functional connectivity. Sci Rep.

[27] Norrie J, Heitger M, Leathem J, Anderson T, Jones R, Flett R (2010). Mild traumatic brain injury and fatigue: a prospective longitudinal study. Brain Inj.

[28] Ouellet MC, Morin CM (2006). Fatigue following traumatic brain injury: Frequency, characteristics, and associated factors. Rehabil Psychol.

[29] Ponsford J, Schonberger M, Rajaratnam SM (2015). A model of fatigue following traumatic brain injury. J Head Trauma Rehabil.

[30] Ponsford JL, Ziino C, Parcell DL, Shekleton JA, Roper M, Redman JR, Phipps-Nelson J, Rajaratnam SM (2012). Fatigue and sleep disturbance following traumatic brain injury–their nature, causes, and potential treatments. J Head Trauma Rehabil.

[31] Potter S, Leigh E, Wade D, Fleminger S (2006). The Rivermead post concussion symptoms questionnaire: a confirmatory factor analysis. J Neurol.

[32] Royston P (2004) Multiple imputation of missing values. Stata J227–241

[33] Schiehser DM, Delano-Wood L, Jak AJ, Hanson KL, Sorg SF, Orff H, Clark AL (2017). Predictors of cognitive and physical fatigue in post-acute mild-moderate traumatic brain injury. Neuropsychol Rehabil.

[34] Steyerberg EW, Wiegers E, Sewalt C, Buki A, Citerio G, De Keyser V, Ercole A, Kunzmann K, Lanyon L, Lecky F, Lingsma H, Manley G, Nelson D, Peul W, Stocchetti N, Von SN, Vande VT, Verheyden J, Wilson L, Maas AIR, Menon DK (2019). Case-mix, care pathways, and outcomes in patients with traumatic brain injury in CENTER-TBI: a European prospective, multicentre, longitudinal, cohort study. Lancet Neurol.

[35] Stulemeijer M, van der Werf S, Bleijenberg G, Biert J, Brauer J, Vos PE (2006). Recovery from mild traumatic brain injury: a focus on fatigue. J Neurol.

[36] Teasdale G, Jennett B (1974). Assessment of coma and impaired consciousness. A practical scale. Lancet.

[37] Voormolen DC, Cnossen MC, Polinder S, Gravesteijn BY, von Steinbuechel N, Real RGL, Haagsma JA (2019). Prevalence of post-concussion-like symptoms in the general population in Italy, The Netherlands and the United Kingdom. Brain Inj.

[38] Voormolen DC, Cnossen MC, Polinder S, von Steinbuechel N, Vos PE, Haagsma JA (2018). Divergent classification methods of post-concussion syndrome after mild traumatic brain injury: prevalence rates, risk factors, and functional outcome. J Neurotrauma.

[39] Voormolen DC, Haagsma JA, Polinder S, Maas AIR, Steyerberg EW, Vulekovic P, Sewalt CA, Gravesteijn BY, Covic A, Andelic N, Plass AM, von Steinbuechel N (2019). Post-concussion symptoms in complicated vs uncomplicated mild traumatic brain injury patients at three and six months post-injury: results from the CENTER-TBI Study. J Clin Med.

[40] Whitehead L (2009). The measurement of fatigue in chronic illness: a systematic review of unidimensional and multidimensional fatigue measures. J Pain Symptom Manage.

[41] Ziino C, Ponsford J (2005). Measurement and prediction of subjective fatigue following traumatic brain injury. J Int Neuropsychol Soc.

