# Correction to: Frequency of fatigue and its changes in the first 6 months after traumatic brain injury: results from the CENTER-TBI study

**DOI:** 10.1007/s00415-020-10174-1

**Published:** 2020-09-10

**Authors:** Nada Andelic, Cecilie Røe, Cathrine Brunborg, Marina Zeldovich, Marianne Løvstad, Daniel Løke, Ida M. Borgen, Daphne C. Voormolen, Emilie I. Howe, Marit V. Forslund, Hilde M. Dahl, Nicole von Steinbuechel, CENTER-TBI participants investigators, Cecilia Åkerlund, Krisztina Amrein, Nada Andelic, Lasse Andreassen, Audny Anke, Anna Antoni, Gérard Audibert, Philippe Azouvi, Maria Luisa Azzolini, Ronald Bartels, Pál Barzó, Romuald Beauvais, Ronny Beer, Bo-Michael Bellander, Antonio Belli, Habib Benali, Maurizio Berardino, Luigi Beretta, Morten Blaabjerg, Peter Bragge, Alexandra Brazinova, Vibeke Brinck, Joanne Brooker, Camilla Brorsson, Andras Buki, Monika Bullinger, Manuel Cabeleira, Alessio Caccioppola, Emiliana Calappi, Maria Rosa Calvi, Peter Cameron, Guillermo Carbayo Lozano, Marco Carbonara, Simona Cavallo, Giorgio Chevallard, Arturo Chieregato, Giuseppe Citerio, Iris Ceyisakar, Hans Clusmann, Mark Coburn, Jonathan Coles, Jamie D Cooper, Marta Correia, Amra Čović, Nicola Curry, Endre Czeiter, Marek Czosnyka, Claire Dahyot-Fizelier, Paul Dark, Helen Dawes, Véronique Keyser, Vincent Degos, Francesco Della Corte, Hugo Boogert, Bart Depreitere, Đula Đilvesi, Abhishek Dixit, Emma Donoghue, Jens Dreier, Guy -Loup Dulière, Ari Ercole, Patrick Esser, Erzsébet Ezer, Martin Fabricius, Valery L Feigin, Kelly Foks, Shirin Frisvold, Alex Furmanov, Pablo Gagliardo, Damien Galanaud, Dashiell Gantner, Guoyi Gao, Pradeep George, Alexandre Ghuysen, Lelde Giga, Ben Glocker, Jagoš Golubovic, Pedro A Gomez, Johannes Gratz, Benjamin Gravesteijn, Francesca Grossi, Russell L Gruen, Deepak Gupta, Juanita A Haagsma, Iain Haitsma, Raimund Helbok, Eirik Helseth, Lindsay Horton, Jilske Huijben, Peter J Hutchinson, Bram Jacobs, Stefan Jankowski, Mike Jarrett, Ji -yao Jiang, Faye Johnson, Kelly Jones, Mladen Karan, Angelos G Kolias, Erwin Kompanje, Daniel Kondziella, Evgenios Koraropoulos, Lars -Owe Koskinen, Noémi Kovács, Ana Kowark, Alfonso Lagares, Linda Lanyon, Steven Laureys, Fiona Lecky, Didier Ledoux, Rolf Lefering, Valerie Legrand, Aurelie Lejeune, Leon Levi, Roger Lightfoot, Hester Lingsma, Andrew I.R. Maas, Ana M Castaño-León, Marc Maegele, Marek Majdan, Alex Manara, Geoffrey Manley, Costanza Martino, Hugues Maréchal, Julia Mattern, Catherine McMahon, Béla Melegh, David Menon, Tomas Menovsky, Ana Mikolic, Benoit Misset, Visakh Muraleedharan, Lynnette Murray, Ancuta Negru, David Nelson, Virginia Newcombe, Daan Nieboer, József Nyirádi, Otesile Olubukola, Matej Oresic, Fabrizio Ortolano, Aarno Palotie, Paul M Parizel, Jean -François Payen, Natascha Perera, Vincent Perlbarg, Paolo Persona, Wilco Peul, Anna Piippo-Karjalainen, Matti Pirinen, Horia Ples, Suzanne Polinder, Inigo Pomposo, Jussi P Posti, Louis Puybasset, Andreea Radoi, Arminas Ragauskas, Rahul Raj, Malinka Rambadagalla, Jonathan Rhodes, Sylvia Richardson, Sophie Richter, Samuli Ripatti, Saulius Rocka, Cecilie Roe, Olav Roise, Jonathan Rosand, Jeffrey V Rosenfeld, Christina Rosenlund, Guy Rosenthal, Rolf Rossaint, Sandra Rossi, Daniel Rueckert, Martin Rusnák, Juan Sahuquillo, Oliver Sakowitz, Renan Sanchez-Porras, Janos Sandor, Nadine Schäfer, Silke Schmidt, Herbert Schoechl, Guus Schoonman, Rico Frederik Schou, Elisabeth Schwendenwein, Charlie Sewalt, Toril Skandsen, Peter Smielewski, Abayomi Sorinola, Emmanuel Stamatakis, Simon Stanworth, Robert Stevens, William Stewart, Ewout W. Steyerberg, Nino Stocchetti, Nina Sundström, Anneliese Synnot, Riikka Takala, Viktória Tamás, Tomas Tamosuitis, Mark Steven Taylor, Braden Te Ao, Olli Tenovuo, Alice Theadom, Matt Thomas, Dick Tibboel, Marjolein Timmers, Christos Tolias, Tony Trapani, Cristina Maria Tudora, Peter Vajkoczy, Shirley Vallance, Egils Valeinis, Zoltán Vámos, Mathieu Jagt, Gregory Steen, Joukje van der Naalt, T.J.M. van Dijck Jeroen, Thomas A. Essen, Wim Hecke, Caroline Heugten, Dominique Praag, Thijs Vyvere, P. J. RoelWijk, Alessia Vargiolu, Emmanuel Vega, Kimberley Velt, Jan Verheyden, Paul M Vespa, Anne Vik, Rimantas Vilcinis, Victor Volovici, Nicole Steinbüchel, Daphne Voormolen, Petar Vulekovic, Kevin K.W. Wang, Eveline Wiegers, Guy Williams, Lindsay Wilson, Stefan Winzeck, Stefan Wolf, Zhihui Yang, Peter Ylén, Alexander Younsi, Frederick A. Zeiler, Veronika Zelinkova, Agate Ziverte, Tommaso Zoerle

**Affiliations:** 1 Department of Physical Medicine and Rehabilitation, Oslo University Hospital, Oslo, Norway; 2 Faculty of Medicine, Institute of Health and Society, Research Centre for Habilitation and Rehabilitation Models and Services (CHARM), University of Oslo, Oslo, Norway; 3 Faculty of Medicine, Institute of Clinical Medicine, University of Oslo, Oslo, Norway; 4 Oslo Centre for Biostatistics and Epidemiology, Oslo University Hospital, Oslo, Norway; 5 Institute of Medical Psychology and Medical Sociology, University Medical Center, Göttingen, Germany; 6 Research Department, Sunnaas Rehabilitation Hospital, Bjørnemyr, Norway; 7 Department of Psychology, Faculty of Social Sciences, University of Oslo, Oslo, Norway; 8 Department of Public Health, Erasmus MC, University Medical Center, Rotterdam, The Netherlands; 9 Department of Child Neurology, Oslo University Hospital, Oslo, Norway

## Correction to: Journal of Neurology 10.1007/s00415-020-10022-2

The original version of this article unfortunately contained a mistake. The Collaborator name Joukje van der Naalt is incorrectly tagged. Therefore, author name is cited incorrectly in PubMed. The given name and family name should be as follows:

Given name: Joukje.

Family name: van der Naalt.

