# Supplementary material for: Frequency of fatigue and its changes in the first 6 months after traumatic brain injury: results from the CENTER-TBI study
Source: J Neurol. 2020 Jul 16;268(1):61–73. doi: 10.1007/s00415-020-10022-2 (PMC7815577; doi:10.1007/s00415-020-10022-2)
Supplement: Supplementary file 1 — Supplementary file1 (DOCX 67 kb) [file 415_2020_10022_MOESM1_ESM.docx]

**Supplement**

**Frequency of fatigue and its changes in the first six months after traumatic brain injury: Results from the CENTER-TBI study**

Authors: Nada Andelic, Cecilie Røe, Cathrine Brunborg, Marina Zeldovich, Marianne Løvstad, Daniel Løke, Ida M. Borgen, Daphne C. Voormolen, Emilie I. Howe, Marit V. Forslund, Hilde M. Dahl, Nicole von Steinbuechel, and CENTER-TBI participants and investigators.

This supplementary material has been provided by the authors to give readers additional information about their work.

**eTable 1** Fatigue severity scores at baseline by age groups and patient strata.

**eFigure 1a** Estimated proportions of patients with fatigue ≥2 by GCS severity levels

**eFigure 1b** Estimated proportions of patients with Fatigue ≥3 by GCS severity levels.

**eTable 2** Changes of fatigue by GCS severity levels. Probabilities are estimated from mixed-effects logistic regression.

**eFigure 2** Main effect and time interaction of feeling depressed on fatigue changes.

**eFigure 3** Main effect and time interaction of sleeping problems on fatigue changes.

**eTable 1** Fatigue severity scores at baseline by age groups and patient strata

|  |  |  |  |  |
| --- | --- | --- | --- | --- |
| **Pat. strata and severity of fatigue** | 0-18 years | 19- 40 years | 41-65 years | >65 years |
| **ER** |  |  |  |  |
| Median (IQR) | 1 (0, 2) | 1 (0, 2) | 0 (0, 2) | 0 (0, 1.5) |
| **Severity** |  |  |  |  |
| None (0-1) | 22 (56.4%) | 140 (52.2%) | 165 (61.3%) | 127 (75.1%) |
| Mild problem (2) | 12 (30.8%) | 68 (25.4%) | 57 (21.2%) | 23 (13.6%) |
| Moderate to severe problem (3-4) | 5 (12.8%) | 60 (22.4%) | 47 (17.5%) | 19 (11.2%) |
| **ADM** |  |  |  |  |
| Median (IQR) | 2 (0, 3) | 2 (0, 2) | 1 (0, 2) | 1 (0, 2) |
| **Severity** |  |  |  |  |
| None (0-1) | 36 (41.9%) | 139 (46.8%) | 223 (52.3%) | 194 (58.3%) |
| Mild problem (2) | 20 (23.3%) | 86 (29.0%) | 98 (23.0%) | 81 (24.3%) |
| Moderate to severe problem (3-4) | 30 (34.9%) | 72 (24.2%) | 105 (24.6%) | 58 (17.4%) |
| **ICU** |  |  |  |  |
| Median (IQR) | 2 (1, 3) | 2 (0, 3) | 2 (0, 3) | 1 (0, 2) |
| **Severity** |  |  |  |  |
| None (0-1) | 11 (26.8%) | 52 (40.9%) | 65 (42.5%) | 41 (52.6%) |
| Mild problem (2) | 10 (24.4%) | 34 (26.8%) | 40 (26.1%) | 20 (25.6%) |
| Moderate to severe problem (3-4) | 20 (48.8%) | 41 (32.3%) | 48 (31.4%) | 17 (21.8%) |

Abbreviations: ER, emergency room; ADM, admission; ICU, intensive care unit; IQR, interquartile range;

**eFigure 1a** Estimated proportions of patients with fatigue ≥2 by GCS severity levels

Note: GCS, Glasgow Coma Scale; Mild TBI = GCS 13-15; Moderate TBI = GCS 9-12; Severe TBI = GCS 3-8;

**eFigure 1b** Estimated proportions of patients with fatigue ≥3 by GCS severity levels

Note: Mild TBI = GCS 13-15; Moderate TBI = GCS 9-12; Severe TBI = GCS 3-8;

**eTable 2** Changes of fatigue by GCS severity levels. Probabilities are estimated from mixed-effects logistic regression.

|  | Baseline (N=2245) | | 3 months (N=2094) | | 6 months (N=2186) | | Within group differences. Baseline to 6 months. (95% CI)  (lower to upper) p-value | Between group difference, (95% CI), p-value |
| --- | --- | --- | --- | --- | --- | --- | --- | --- |
|  | n | p (95% CI) | n | p (95% CI) | n | p (95% CI) |  |  |
| **Fatigue ≥2** |  |  |  |  |  |  |  |  |
| Mild TBI  (GCS 13-15) | 959 | 46.7 (44.5 to 48.8) | 710 | 43.8 (41.4 to 46.1) | 699 | 41.7 (39.4 to 44.0) | -5.0 (-7.8 to -2.2), p<0.001 | Ref. |
| Moderate TBI  (GCS 9-12) | 38 | 60.5 (49.1 to 72.0) | 91 | 60.7 (53.0 to 68.4) | 94 | 53.0 (45.6 to 60.5) | -7.5 (-20.3 to 5.3), p=0.250 | -2.5 (-15.6 to 10.6), p=0.706 |
| Severe TBI  (GCS 3-8) | 53 | 65.8 (56.6 to 75.1) | 214 | 67.2 (62.0 to 72.3) | 218 | 62.8 (57.7 to 67.8) | -3.1 (-13.0 to 6.9), p=0.548 | 1.9 (-8.4 to 12.3), p=0.715 |
| **Fatigue ≥3** |  |  |  |  |  |  |  |  |
| Mild TBI  (GCS 13-15) | 131 | 22.6 (20.8 to 24.3) | 73 | 23.1 (21.1 to 25.1) | 83 | 22.3 (20.3 to 24.2) | -0.3 (-2.7 to 2.0), p=0.808 | Ref. |
| Moderate TBI  (GCS 9-12) | 265 | 35.8 (24.6 to 47.1) | 201 | 34.4 (26.9 to 41.9) | 186 | 31.8 (25.0 to 38.6) | -4.0 (-16.5 to 8.4), p=0.527 | -3.7 (-16.4 to 8.9), p=0.564 |
| Severe TBI  (GCS 3-8) | 126 | 39.9 (30.1 to 49.7) | 290 | 37.0 (31.8 to 42.3) | 303 | 33.4 (28.5 to 38.3) | -6.5 (-16.9 to 3.9), p=0.220 | -6.2 (-16.9 to 4.5), p=0.254 |

n: number of participants with values above cut-off scores; p = probability of fatigue; Mild TBI = GCS 13-15; Moderate TBI = GCS 9-12; Severe TBI = GCS 3-8;

**eFigure 2**. Main effect and time interaction of feeling depressed on fatigue changes.

**eFigure 3.** Main effect and time interaction of sleeping problems on fatigue changes.
